# Supplementary material for: Modelling Skylarks (Alauda arvensis) to Predict Impacts of Changes in Land Management and Policy: Development and Testing of an Agent-Based Model
Source: PLoS One. 2013 Jun 6;8(6):e65803. doi: 10.1371/journal.pone.0065803 (PMC3675089; doi:10.1371/journal.pone.0065803)
Supplement: Supporting Information S4 — The skylark ODdox as a zipped archive. (ZIP) [file pone.0065803.s004.zip › Skylark_ODdox/struct_rotation.html]

ALMaSS Skylark ODdox: Rotation Struct Reference


ALMaSS Skylark ODdox
 2.0


- Main Page
- Related Pages
- Classes
- Files

- Class List
- Class Index
- Class Hierarchy
- Class Members

Public Attributes

Rotation Struct Reference

`#include <croprotation.h>`

List of all members.

|  |  |
| --- | --- |
| Public Attributes | |
| TTypesOfVegetation | CropNum [NoFarmTypes] |
| TTypesOfVegetation | NewCrop [NoFarmTypes][4] |
| int | Percent [NoFarmTypes][4] |

---

## Member Data Documentation

TTypesOfVegetation Rotation::CropNum[NoFarmTypes]

TTypesOfVegetation Rotation::NewCrop[NoFarmTypes][4]

int Rotation::Percent[NoFarmTypes][4]

---

The documentation for this struct was generated from the following file:

- croprotation.h


- Rotation
- Generated on Thu Jan 10 2013 13:15:37 for ALMaSS Skylark ODdox by
   1.8.1.1
